# Identification of a Novel Nomogram to Predict Progression Based on the Circadian Clock and Insights Into the Tumor Immune Microenvironment in Prostate Cancer

**DOI:** 10.3389/fimmu.2022.777724

**Published:** 2022-01-27

**Authors:** Dechao Feng, Qiao Xiong, Facai Zhang, Xu Shi, Hang Xu, Wuran Wei, Jianzhong Ai, Lu Yang

**Affiliations:** Department of Urology, Institute of Urology, West China Hospital, Sichuan University, Chengdu, China

**Keywords:** circadian rhythm, circadian clock, gene signature, tumor immune microenvironment, predictive model, targeted therapy

## Abstract

**Background:**

Currently, the impact of the circadian rhythm on the tumorigenesis and progression of prostate cancer (PCA) has yet to be understood. In this study, we first established a novel nomogram to predict PCA progression based on circadian clock (CIC)-related genes and provided insights into the tumor immune microenvironment.

**Methods:**

The TCGA and Genecards databases were used to identify potential candidate genes. Lasso and Cox regression analyses were applied to develop a CIC-related gene signature. The tumor immune microenvironment was evaluated through appropriate statistical methods and the GSCALite database.

**Results:**

Ten genes were identified to construct a gene signature to predict progression probability for patients with PCA. Patients with high-risk scores were more prone to progress than those with low-risk scores (hazard ratio (HR): 4.11, 95% CI: 2.66-6.37; risk score cut-off: 1.194). CLOCK, PER (1, 2, 3), CRY2, NPAS2, RORA, and ARNTL showed a higher correlation with anti-oncogenes, while CSNK1D and CSNK1E presented a greater relationship with oncogenes. Overall, patients with higher risk scores showed lower mRNA expression of PER1, PER2, and CRY2 and higher expression of CSNK1E. In general, tumor samples presented higher infiltration levels of macrophages, T cells and myeloid dendritic cells than normal samples. In addition, tumor samples had higher immune scores, lower stroma scores and lower microenvironment scores than normal samples. Notably, patients with higher risk scores were associated with significantly lower levels of neutrophils, NK cells, T helper type 1, and mast cells. There was a positive correlation between the risk score and the tumor mutation burden (TMB) score, and patients with higher TMB scores were more prone to progress than those with lower TMB scores. Likewise, we observed similar results regarding the correlation between the microsatellite instability (MSI) score and the risk score and the impact of the MSI score on the progression-free interval. We observed that anti-oncogenes presented a significantly positive correlation with PD-L1, PD-L2, TIGIT and SIGLEC15, especially PD-L2.

**Conclusion:**

We identified ten prognosis-related genes as a promising tool for risk stratification in PCA patients from the fresh perspective of CIC.

## Introduction

Prostate cancer (PCA) is a heterogeneous disease and ranks as the fifth leading cause of male cancer-related deaths worldwide, with an estimated figure of almost 1.4 million new cases and 375,000 deaths in 2020 (1). On a global scale, the age-standardized incidence rate of PCA increased from 30.5 cases per 100,000 population in 1990 to 37.9 cases per 100,000 population in 2017, which is positively related to the sociodemographic index in most regions (2). The PCA incidence has been increasing in most Asian countries, ranking first in Chinese men regarding its incidence and mortality among all urologic tumors (1, 3). This increase might be attributed to not only increased PSA screening but also the conversion of a westernized lifestyle (4). The management of PCA is characterized by diversity and complexity, in which radical prostatectomy, radiotherapy, and androgen deprivation therapy are important components to the treatment of organ-localized and androgen-dependent PCA (5). However, approximately one-third of patients suffer from recurrence after localized treatment and eventually progress to castration-resistant PCA, which is a predominant contributor to death in PCA patients (6).

Circadian rhythm (CDH) has been proven to be involved in a variety of behavioral and physiological processes in mammals, such as the sleep-wake cycle, cognitive function, blood pressure, heart rate, and hormone secretion, which are controlled by the pacemaker and its regulator (7, 8). The former is located at the suprachiasmatic nuclei (SCN) in the anterior hypothalamus, while the latter consists of clock genes expressed in central circadian pacemakers as well as many peripheral cells and tissues (9–11). The known key genes for the circadian clock (CIC) include CLOCK, ARNTL (also named BMAL1), Period (PER1, PER2, PER3), Cryptochrome (Cry1, Cry2), NPAS2, NR1D1 (also named REV-ERBA), NR1D2 (also named REV-ERBB), RORA, CSNK1D and CSNK1E (12). Desynchronization of the CDH plays a pivotal role in carcinogenesis and the establishment of cancer hallmarks (13).

Precision medicine has been an important concept in multidisciplinary areas, while illuminating the mechanism of tumorigenesis and tumor progression at the gene level is attracting considerable critical attention. It has been previously observed that genetic variants of CIC genes might be related to PCA progression (14–18). However, so far, there has been little agreement on whether night shift work is associated with increased PCA risk (19–21). Thus, we proposed that CIC mainly promotes PCA development and progression through indirect mechanisms, such as androgen receptor (AR) (7), epithelial-mesenchymal transition and the tumor microenvironment (22, 23). In this study, we firstly established a novel nomogram to predict PCA progression based on CIC-related genes and provided insights into the tumor immune microenvironment.

## Methods

### Data Preparation

All somatic mutation data, raw counts of RNA-sequencing data (level 3) and corresponding clinical information of PCA were obtained from The Cancer Genome Atlas (TCGA) database (https://portal.gdc.cancer.gov/), and the method of acquisition and application complied with the guidelines and policies. The RNA-seq data in the format of fregments per kilobase per million (FPKM) were converted to the format of transcripts per million reads (TPM), and log2 conversion was performed (24). The prognostic data were integrated into the analyzed dataset (25). Genes with a false discovery rate (FDR)-adjusted p value < 0.001 and an absolute value of log2 (fold change) > 1 were considered differentially expressed genes (DEGs). Genes associated with progression-free interval (PFI) were defined as an adjusted p value < 0.05 through Cox regression analysis. We used PFI as a prognostic factor, which was defined as the period during and after treatment in which a participant was living with a disease that did not worsen. Usually, it is the period from date of diagnosis until 1) locoregional or systemic recurrence, 2) second malignancy, or 3) death from any cause; late deaths not related to cancer or its treatment are excluded (25). The CIC-related genes were obtained from Genecards (https://www.genecards.org/) (26). Somatic mutations included nonsilence mutations, nonsynonymous mutations, deletion mutations, frameshift mutations, insertion mutations and so on. Tumor mutation burden (TMB) is defined by the number of somatic mutations (in addition to synonymous mutations and intron mutations) per genome area (38 Mb) for target sequencing (27). Microsatellite instability (MSI) is a pattern of hypermutation that occurs at genomic microsatellites and is caused by defects in the mismatch repair system (28). MSI data were obtained from R package “TCGAbiolinks”. The specific classification and description of markers in 24 immune cells were performed by the previous article (29).

### Analysis of HPA, cBioPortal, and GSCALite

Validation of candidate genes at the protein level was performed through the HPA database (https://www.proteinatlas.org/) (30–32). The genetic alteration and mutation location of the ten genes were derived from cBioPortal (http://www.cbioportal.org/; TCGA, PanCancer Atlas; RNA Seq V2 RSEM) (33, 34). The GSCALite database was used to further analyze mutation information, immune infiltration, and gene set variation analysis (GSVA) of enrolled genes (http://bioinfo.life.hust.edu.cn/web/GSCALite/) (35). In addition, we explored the Genomics of Drug Sensitivity in Cancer (GDSC) and the Cancer Therapeutics Response Portal (CTRP) drug sensitivity in pan cancer through GSCALite (35).

### Functional Enrichment Analysis

Gene ontology (GO) and Kyoto Encyclopedia of Genes and Genome (KEGG) analyses of each candidate gene and gene set were conducted to explore possible biological functions and signaling pathways using the package R “clusterProfiler”. GO analysis included biological process (BP), cell composition (CC) and molecular function (MF) (P<0.05 was statistically significant).

### Statistical Analysis

All analyses were conducted with R version 3.6.3 (https://www.r-project.org/) and its suitable packages. Principal component analysis was performed by the R package “Factoextra” to nonlinear dimensionality reduction and cluster analysis. To make reliable immune infiltration estimations, we utilized the R package “immunedeconv”, including xCell, MCP-counter, and CIBERSORT (36–42). The ssGSEA algorithm included in the R package “GSVA” was also used to assess the immune infiltration level (43). The immune score was imputed by using the “estimate” package. SIGLEC15, TIGIT, CD274, HAVCR2, PDCD1, CTLA4, LAG3, and PDCD1LG2 were selected as immune checkpoint-relevant transcripts, and the expression values of these eight genes were extracted (44–47). Lasso Cox regression modeling was conducted by using the “glmnet” package. A predictive nomogram was further constructed based on Cox regression analysis. The chi-square test was used to assess differences between categorical variables, and t tests or paired sample t tests were used for continuous variables. The two-gene correlation map was realized by the R package “ggstatsplot”, and the multigene correlation map was displayed by the R package “pheatmap”. We used Spearman’s correlation analysis to describe the correlation between quantitative variables without a normal distribution. The survival analysis was conducted through Kaplan–Meier curves and log-rank tests. All the statistical tests mentioned above are two-sided. P values of < 0.05 were considered statistically significant. Distinctive mark: no significance (ns), p≥0.05; *, p< 0.05; **, p<0.01; ***, p<0.001.

## Results

### Baseline Data and Clinical Values

**Figure 1:**
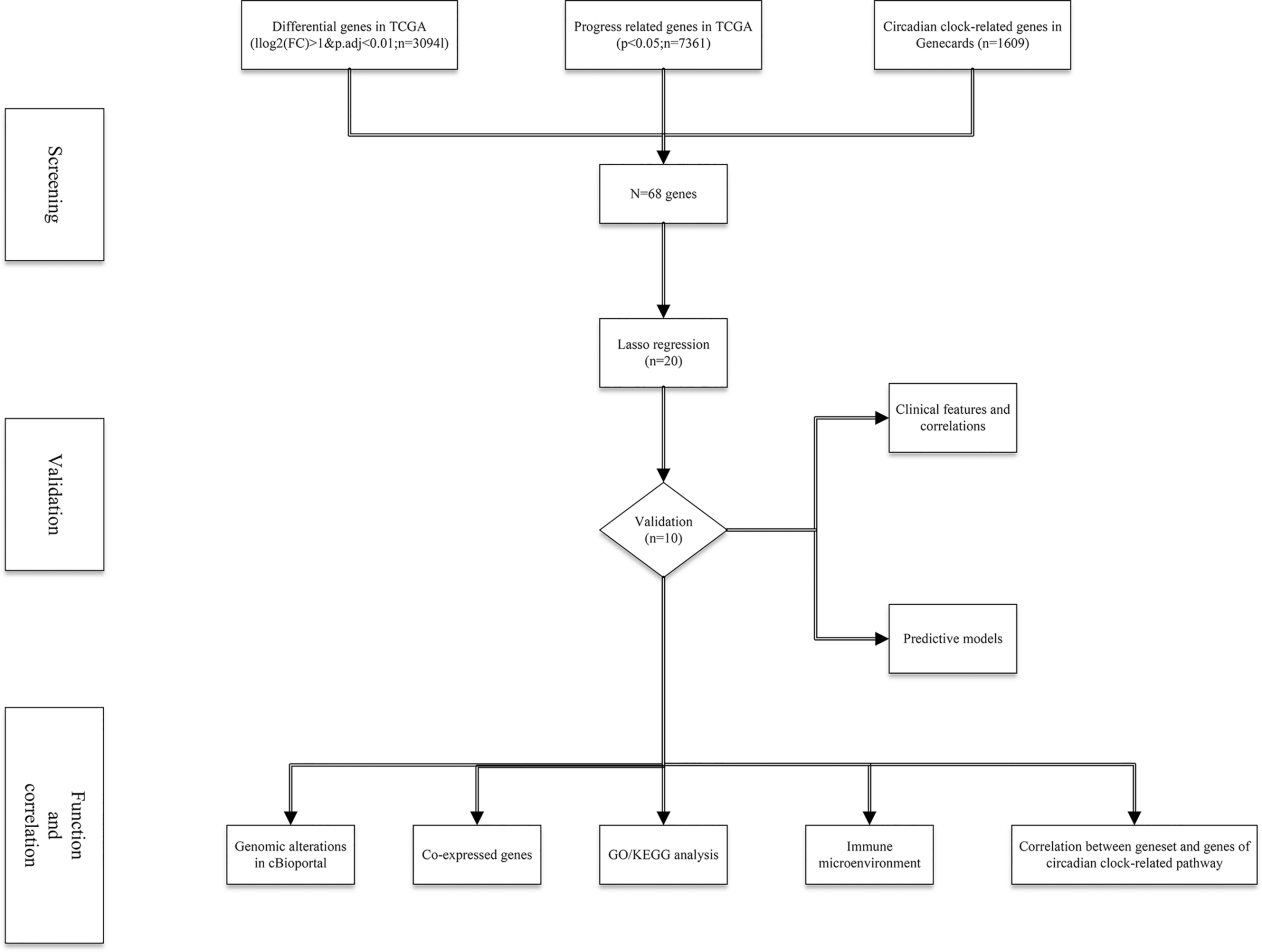
The flow chart of this study.

**Figure 2:**
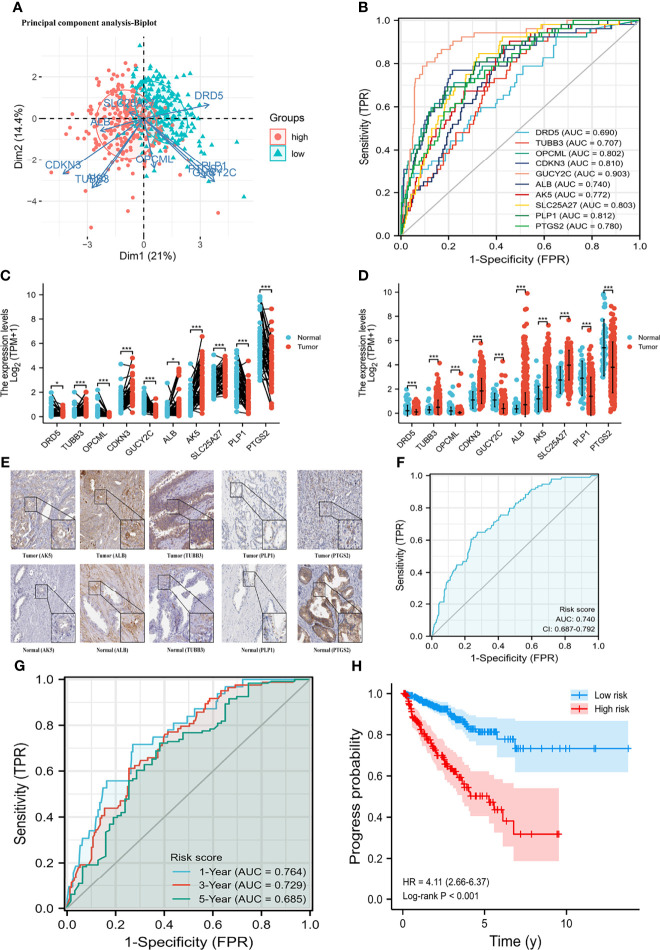
Differential mRNA expression and clinical values of enrolled genes. **(A)** principal component analysis-Biplot; **(B)** the receiver operating characteristic curves of genes; **(C)** gene differential expression in paired samples; **(D)** gene differential expression in non-paired samples; **(E)** gene differential expression at protein level; **(G)** time-dependent receiver operating characteristic curve of risk score; **(H)** Kaplan-Meier curve of two risk groups. HR, hazard ratio; AUC, area under curve; CI, confidence interval. **(F)** the receiver operating characteristic curve of risk score for prostate cancer progression. *means smaller than 0.01; **means smaller than 0.01; ***means smaller than 0.001.

A total of 498 tumor and 52 adjacent normal tissues in the TCGA database were analyzed. Sixty-eight genes were identified after the intersection of CIC-related genes in Genecards (26) and differential and progression-related genes in TCGA. Subsequently, Lasso regression analysis was used to identify 20 candidate genes. Finally, 10 genes were used to establish a predictive model by validating the consistency of DEGs and prognosis. A flow chart of this study was provided in **Figure 1**. The results of principal component analysis, indicating consistency of gene function and risk group were presented (**Figure 2A**). We calculated the area under the receiver operating characteristic curve (ROC) of the ten genes, indicating certain accuracy of most genes in predicting tumors and normal tissues (**Figure 2B**). The DEGs at the mRNA level and at the protein level were presented in **Figures 2C–E**. In predicting progress, the risk score of the predictive model showed certain accuracy and stability (**Figures 2F, G**). Moreover, we detected that high-risk patients were more prone to progress than low-risk patients (hazard ratio (HR): 4.11, 95% CI: 2.66-6.37; risk score cut-off: 1.194; **Figure 2H**). CDKN3, AK5, SLC25A27, TUBB3, and ALB showed better diagnostic values and stability in predicting progression as a result of time-dependent ROC curves (**Supplementary Figure 1**). The diagnostic and prognostic values and subgroup survival analysis of the ten genes were detailed in the **Supplementary Material**. The enrolled genes functioned in protein coding. The basic gene information was depicted in **Table 1**. Likewise, the baseline data of the ten genes in PCA patients based on TCGA database were presented in **Table 2**. Overall, the differential expression of most genes was related to T stage and Gleason score.

**Table 1:** The basic information of investigative genes in this study.

Gene Symbol	Gene description	Gene biotype	Subcellular locations from UniprotKB/Swiss-prot	Function	Relevance scores from Genecards
DRD5	Dopamine Receptor D5	Protein coding	Cell membrane. Multi-pass membrane protein	Anti-oncogene	0.891700923
TUBB3	Tubulin Beta 3 Class III	Protein coding	Cytoplasm, cytoskeleton. Cell projection, growth cone. Cell projection, lamellipodium. Cell projection, filopodium	Oncogene	1.114164233
OPCML	Opioid Binding Protein/Cell Adhesion Molecule Like	Protein coding	Cell membrane. Lipid-anchor, Glycosylphosphatidylinositol-anchor	Anti-oncogene	1.546406031
CDKN3	Cyclin Dependent Kinase Inhibitor 3	Protein coding	Cytoplasm, perinuclear region	Oncogene	2.472492695
GUCY2C	Guanylate Cyclase 2C	Protein coding	Cell membrane	Anti-oncogene	1.649936557
ALB	Albumin	Protein coding	Secreted.	Oncogene	4.872463226
AK5	Adenylate Kinase 5	Protein coding	Cytoplasm	Oncogene	0.890497983
SLC25A27	Solute Carrier Family 25 Member 27	Protein coding	Mitochondrion inner membrane. Multi-pass membrane protein.	Oncogene	0.746393979
PLP1	Proteolipid Protein 1	Protein coding	Cell membrane. Multi-pass membrane protein. Myelin membrane.	Anti-oncogene	0.262908012
PTGS2	Prostaglandin-Endoperoxide Synthase 2	Protein coding	Microsome membrane. Peripheral membrane protein. Endoplasmic reticulum membrane. Nucleus inner membrane.	Anti-oncogene	0.658713877

**Table 2:** The basic clinicopathologic characteristics of enrolled genes in PCA patients based on TCGA database.

Features	AK5			ALB			CDKN3			DRD5			GUCY2C		
	Low	High (n=250)	P	Low (n=249)	High (n=250)	P	Low (n=249)	High (n=250)	P	Low (n=249)	High (n=250)	P	Low (n=249)	High (n=250)	P
	(n=249)														
T stage			0.163			0.358			< 0.001			0.036			0.752
T2	104	85		101	88		131	58		81	108		98	91	
T3	135	157		141	151		109	183		158	134		142	150	
T4	5	6		4	7		4	7		7	4		6	5	
N stage			0.047			0.044			< 0.001			< 0.001			1
N0	173	174		178	169		177	170		160	187		170	177	
N1	29	50		30	49		23	56		58	21		39	40	
M stage			1			1			1			0.248			1
M0	227	228		226	229		222	233		227	228		233	222	
M1	2	1		1	2		1	2		3	0		2	1	
Race			0.169			0.779			0.426			0.195			0.326
Asian	4	8		6	6		6	6		9	3		8	4	
Black or African American	24	33		26	31		33	24		30	27		31	26	
White	217	198		210	205		202	213		204	211		200	215	
Age			0.08			0.08			0.022			0.19			0.042
<=60	122	102		122	102		125	99		104	120		100	124	
>60	127	148		127	148		124	151		145	130		149	126	
Zone of origin			0.462			0.653			0.28			0.55			0.327
Central Zone	2	2		2	2		2	2		1	3		3	1	
Overlapping/Multiple Zones	68	58		58	68		46	80		74	52		70	56	
Peripheral Zone	62	75		57	80		62	75		76	61		78	59	
Transition Zone	3	5		2	6		5	3		4	4		7	1	
PSA(ng/ml)			1			0.884			0.031			0.323			1
<4	208	207		214	201		220	195		197	218		204	211	
>=4	14	13		13	14		8	19		16	11		13	14	
Gleason score			0.004			0.013			< 0.001			< 0.001			0.583
6	32	14		30	16		35	11		16	30		27	19	
7	131	116		134	113		152	95		107	140		120	127	
8	31	33		25	39		27	37		36	28		28	36	
9	54	84		58	80		34	104		87	51		72	66	
10	1	3		2	2		1	3		3	1		2	2	
Features	OPCML			PLP1			PTGS2			TUBB3			SLC25A27		
	Low (n=249)	High (n=250)	P	Low (n=249)	High (n=250)	P	Low (n=249)	High (n=250)	P	Low (n=249)	High (n=250)	P	Low (n=249)	High (n=250)	P
T stage			0.081			0.03			0.004			< 0.001			0.526
T2	82	107		80	109		80	109		119	70		97	92	
T3	157	135		158	134		158	134		126	166		141	151	
T4	5	6		7	4		9	2		0	11		7	4	
N stage			0.081			0.15			0.05			< 0.001			0.155
N0	166	181		168	179		157	190		184	163		174	173	
N1	47	32		46	33		46	33		18	61		32	47	
M stage			0.623			0.12			1			0.248			0.621
M0	226	229		224	231		227	228		227	228		230	225	
M1	2	1		3	0		2	1		0	3		1	2	
Race			0.009			0.07			0.091			0.641			0.021
Asian	10	2		9	3		9	3		7	5		6	6	
Black or African American	21	36		23	34		32	25		31	26		19	38	
White	210	205		212	203		197	218		204	211		220	195	
Age			0.342			0			0.555			0.054			0.818
<=60	106	118		95	129		108	116		123	101		110	114	
>60	143	132		154	121		141	134		126	149		139	136	
Zone of origin			0.478			0.06			0.115			0.038			0.049
Central Zone	3	1		2	2		3	1		3	1		1	3	
Overlapping/Multiple Zones	70	56		75	51		60	66		45	81		50	76	
Peripheral Zone	70	67		66	71		72	65		68	69		55	82	
Transition Zone	6	2		7	1		7	1		5	3		7	1	
PSA (ng/ml)			0.413			0.06			1			0.123			0.709
<4	204	211		205	210		200	215		210	205		207	208	
>=4	16	11		19	8		13	14		9	18		15	12	
Gleason score			0.083			0			0.014			< 0.001			< 0.001
6	20	26		21	25		23	23		35	11		25	21	
7	119	128		104	143		107	140		142	105		145	102	
8	26	38		36	28		35	29		36	28		16	48	
9	82	56		86	52		80	58		35	103		60	78	
10	2	2		2	2		4	0		1	3		3	1	

PSA, prostate specific antigen; PCA, prostate cancer; TCGA, the Cancer Genome Atlas.

**Figure 3:**
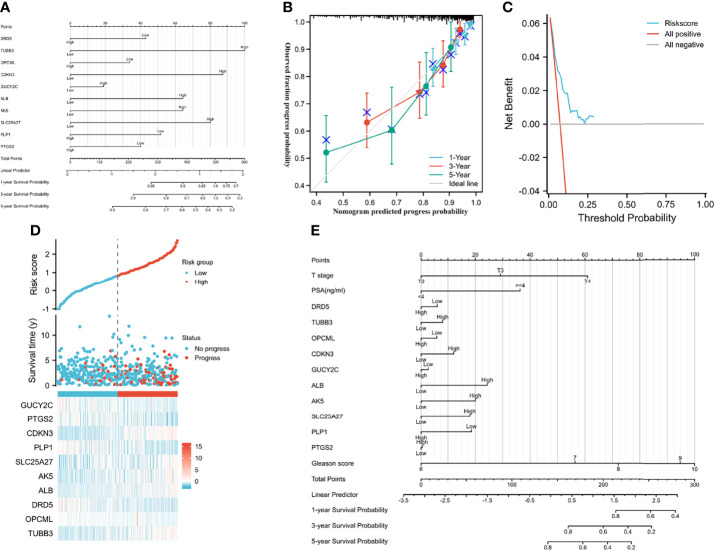
Predictive models and heatmap of risk fator. **(A)** the nomogram of gene signature; **(B)** the calibration plot; **(C)** the result of decision curve analysis; **(D)** heatmap of risk factor; **(E)** the nomogram of gene signature and clinical parameter.

We established a nomogram to predict PFI in PCA patients (concordance (also named C-index): 0.734; 95% CI: 0.707-0.760; **Figure 3A**). **Figures 3B, C** showed the calibration (C-index: 0.734; se: 0.026) and decision curve analysis (C-index: 0.734; 95% CI: 0.707-0.760). The risk score plot indicated that high-risk patients were susceptible to progression compared to low-risk patients (**Figure 3D**). T stage, PSA, and Gleason score were regarded as clinically independent progress factors through the univariable and multivariable Cox regression analyses (**Supplementary Table 1**). We subsequently integrated the clinical factors into the gene nomogram (C-index: 0.777; 95% CI: 0.752-0.802; **Figure 3E**), and no significant improvement was observed in predictive ability.

### Clinical Correlations and Function Analysis

**Figure 4:**
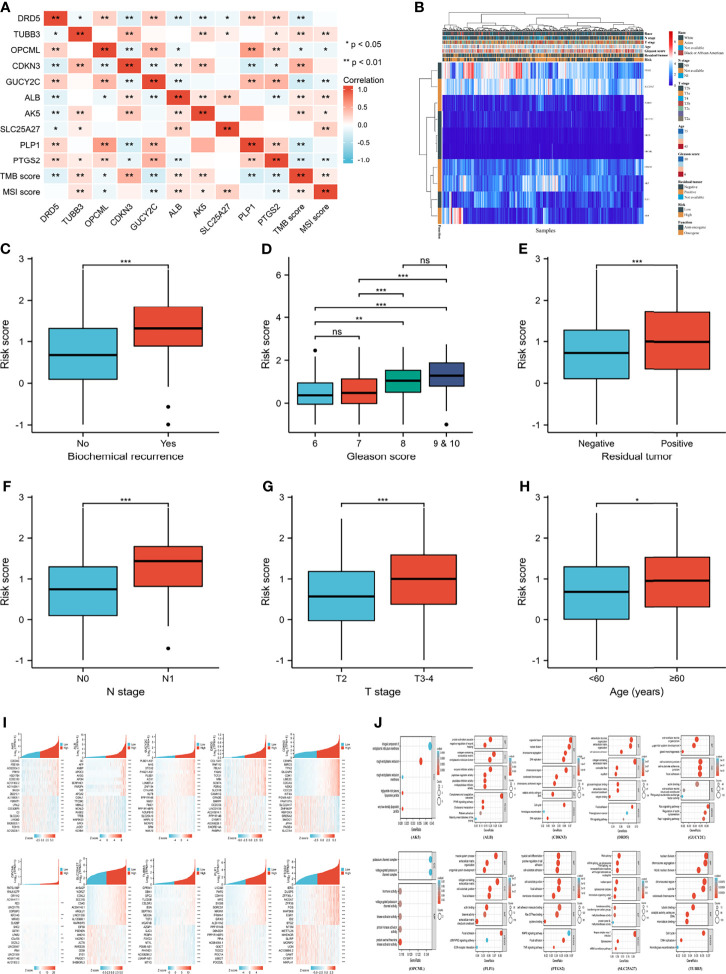
Clinical correlation and function enrichment analysis. **(A)** correlation among genes, TMB score and MSI score; **(B)** the integrated heatmap showing correlation between genes and clinical parameters; **(C)** the correlation between biochemical recurrence and risk score; **(D)** the correlation between Gleason score and risk score; **(E)** the correlation between residual tumor and risk score; **(F)** the correlation between N stage and risk score; **(G)** the correlation between T stage and risk score; **(H)** the correlation between age and risk score; **(I)** the co-expressed genes of candidate genes; **(J)** the Gene ontology and Kyoto Encyclopedia of Genes and Genome analysis of enrolled genes. TMB, tumor mutation burden; MSI, microsatellite instability; BP, biological process; CC, cell composition; MF, molecular function. no significance (ns) means ≥0.05; ***means smaller than 0.001.

**Figure 4A** showed good correlations between genes. Additionally, TMB and MSI were positively associated with oncogenes but negatively related to anti-oncogenes (**Figure 4A**). Correlations among genes, risk score and clinical parameters were summarized in **Figure 4B**. Patients with higher risk scores were prone to biochemical recurrence, higher Gleason scores, positive residual tumors, positive N stages, higher T stages and older age (**Figures 4C–H**). CDKN3 and PLP1 presented a better clinical relationship in the subgroup analysis of PFI. The mRNA expression of CDKN3 tended to rise with increasing Gleason score, N stage, T stage, age, residual tumor, and deterioration of therapy outcome. In contrast, PLP1 mRNA expression decreased with increasing age, Gleason score, and T stage. The specific clinical correlations of the included genes were shown in **Supplementary Figure 2**.

**Figure 4I** showed the heatmap of the most positively and negatively relevant genes of the enrolled genes. The results of GO and KEGG analysis of each gene were shown in **Figure 4J**. The enriched pathways of the gene set included choline metabolism in cancer, the Hippo signaling pathway, and the TGF-beta signaling pathway, and GSVA analysis of the top 20 co-expressed genes of the enrolled genes indicated that PCA was positively related to hormone AR and was negatively associated with epithelial-mesenchymal transition (**Supplementary Figure 3**).

### Genomic Alterations

**Figure 5:**
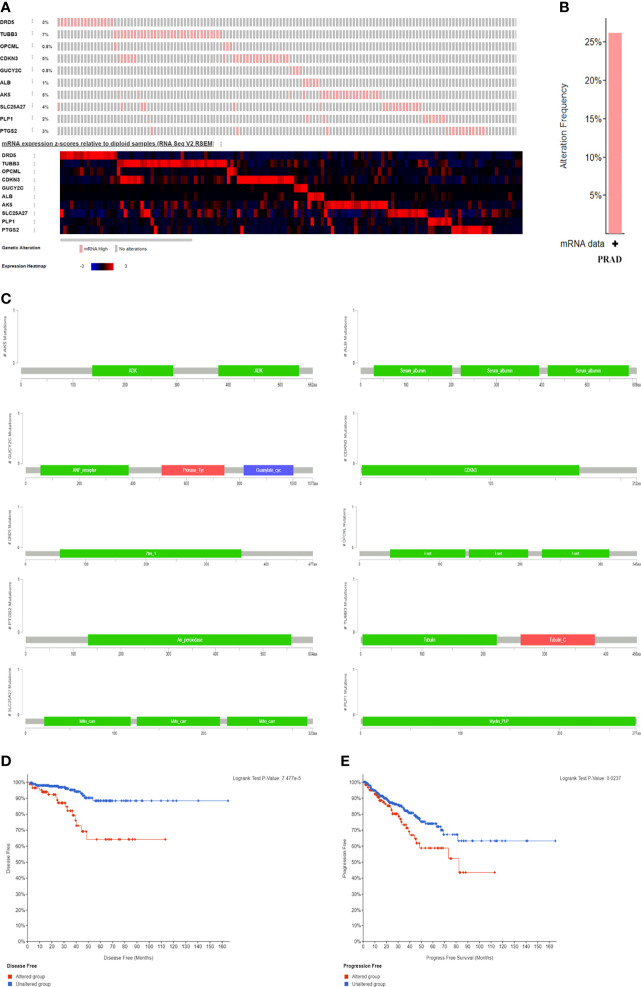
The genomic alterations and survival difference between altered group and unaltered group. **(A)** the mRNA expression frequency of genes; **(B)** the alteration frequency in PCA patients; **(C)** the mutation location of genes; **(D)** the difference of altered and unaltered group regarding disease free survival; **(E)** the difference of altered and unaltered group regarding progress free survival.

**Figure 6:**
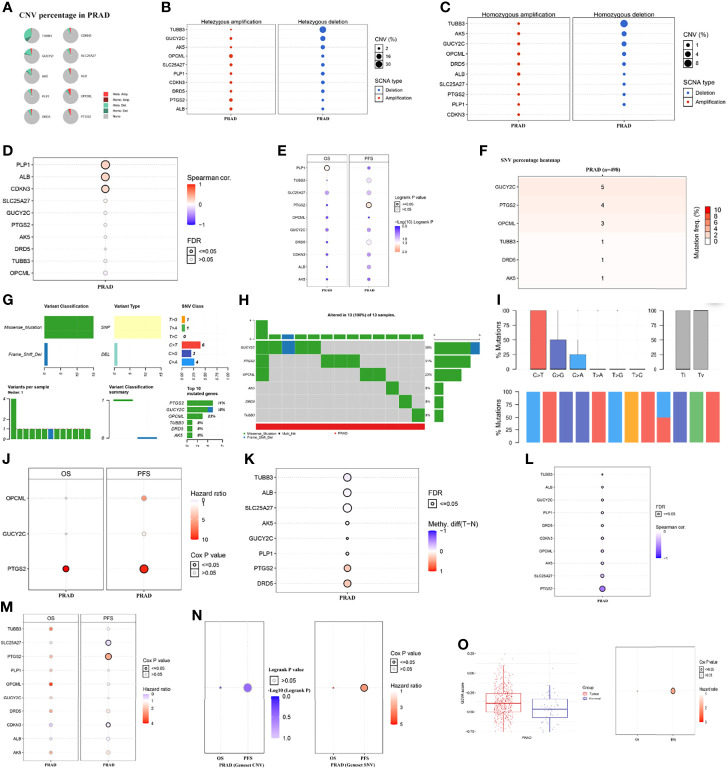
The mutation situation of genes and its correlation with overall survival and progress free survival in PCA patients at the levels of copy number variation, single nucleotide variation, and methylation from GSCALite (35). **(A)** CNV percentage in PCA patients; **(B)** the profile of heterozygous CNV of genes; **(C)** the profile of homozygous CNV of genes; **(D)** the correlations between CNV and mRNA expression; **(E)** the difference of survival between CNV and wide type; **(F)** SNV percentage heatmap; **(G)** SNV classes; **(H)** oncoplot providing the situation of the SNV of mutated genes; **(I)** the Transitions and transversions classification of the SNV of inputted gene set; **(J)** the survival difference between mutant (deleterious) and wide type; **(K)** the methylation difference between tumor and normal samples of inputted genes; **(L)** the profile of correlations between methylation and mRNA expression of inputted genes; **(M)** the overall survival difference between higher and lower methylation groups; **(N)** the survival difference between WT and gene set CNV and SNV; **(O)** the GSVA score of gene set in tumor samples and normal samples and its difference in OS and PFS. CNV, copy number variation; SNV, single nucleotide variation; OS, overall survival; PFS, progress free survival; FDR, false discovery rate.

The genetic alteration and mutation location of the ten genes from cBioPortal (TCGA, PanCancer Atlas; RNA seq V2; 493 patients/samples) were shown in **Figures 5A–C**. Patients in the altered group experienced shorter disease-free survival and PFI than those in the unaltered group (**Figures 5D, E**). **Figure 6** presented the mutation status of genes and its correlation with overall survival (OS) and PFI in PCA patients at the levels of copy number variation (CNV), single nucleotide variation (SNV), and methylation from GSCALite (35). A positive relationship between CNV and mRNA expression was detected in PLP1, ALB and CDKN3 in PCA patients (**Figure 6D**), and CNV of PLP1 and PTGS2 had shorter OS and PFI than wild-type (WT) PLP1 and PTGS2 (**Figure 6E**). The SNVs of the investigated genes were shown in **Figures 6F–J**. GUCY2C, PTGS2, and OPCML showed more effective SNV mutations (**Figure 6F**), and PTGS2 SNV had a higher risk of OS and progression-free survival (PFS) than PTGS2 WT (**Figure 6J**). **Figure 6K** summarized the methylation difference between tumor and normal samples of inputted genes in PCA patients. Higher methylation of DRD5 and PTGS2 and lower methylation of TUBB3, ALB and SLC25A27 were obviously detected. In addition, the mRNA expression of the enrolled genes was negatively associated with methylation (**Figure 6L**). These results were consistent with the functions of oncogenes and anti-oncogenes. Methylation of PTGS2 and demethylation of SLC25A27 and CDKN3 showed lower PFS than their counterparts in PCA patients (**Figure 6M**). In terms of the ten-gene signature, the SNV gene set had a higher risk of PFS (**Figure 6N**) than the WT group. Moreover, the gene set had a higher GSVA score and was prone to progress when compared to a lower GSVA score (**Figure 6O**).

### Tumor Immune Microenvironment and Correlation Between Enrolled Genes and CIC Pathway Genes

**Figure 7:**
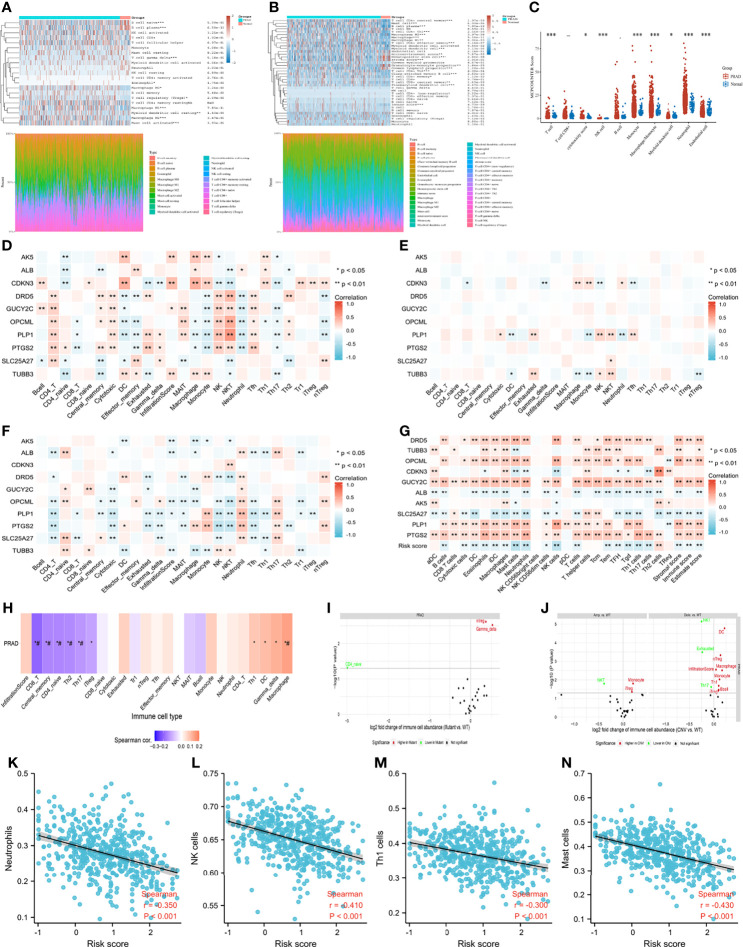
Tumor immune microenvironment. **(A)** immune infiltration estimations using CIBERSORT algorithm; **(B)** immune infiltration estimations using Xcell algorithm; **(C)** immune infiltration estimations using MCP-counter algorithm; **(D)** the correlation between gene mRNA expression and immune cells; **(E)** the correlation between gene CNV and immune cells; **(F)** the correlation between gene methylation and immune cells; **(G)** the correlation between gene mRNA expression and immune cells using ssGSEA algorithm; **(H)** the correlation between gene set GSVA scores and immune cells; **(I)** the correlation between gene set SNV and immune cells; **(J)** the correlation between gene set CNV and immune cells; **(K)** the correlation between risk score and neutrophils; **(L)** the correlation between risk score and natural killer (NK) cells; **(M)** the correlation between risk score and T helper type 1 cells; **(N)** the correlation between risk score and mast cells. ##: false discovery rate ≤ 0.05.

**Figures 7A–C** presented the immune score distribution in PCA tissues and normal tissues using three algorithms (Cibersort, Xcell and Mcpcounter). In general, tumor samples presented higher infiltration levels of macrophages, T cells and myeloid dendritic cells than normal samples. In addition, tumor samples had higher immune scores, lower stroma scores and lower microenvironment scores than normal samples. Correlations between genes and immune infiltration in PCA patients from GSCALite (35) were presented at the mRNA level (**Figure 7D**), CNV level (**Figure 7E**), and methylation level (**Figure 7F**). Overall, natural killer T cells, natural killer (NK) cells and cytotoxic T cells were positively related to tumor suppressor genes, while dendritic cells, macrophages, and monocytes were positively associated with oncogenes (**Figure 7D**). Notably, a positive correlation was observed between natural killer T cells and the mRNA expression of DRD5, OPCML and PLP1. Both dendritic cells and macrophages were positively related to the mRNA expression of AK5 and CDKN3. No obvious relationship was detected between the CNV gene and immune infiltration (**Figure 7E**). Gene methylation had the opposite effect on immune infiltration compared to gene mRNA expression (**Figure 7F**), which was consistent with the correlation between gene expression and methylation (**Figure 6L**). We used ssGSEA to assess the association between the immune infiltration level and genes (**Figure 7G**), resulting in similar results to the above. In general, increased expression of oncogenes and decreased expression of anti-oncogenes were positively related to a decrease in immune infiltration, especially for NK cells, neutrophils, mast cells, macrophages, stromal score, immune score and ESTIMATE score. In terms of the gene set, mRNA expression of gene set was positively related to macrophage, and was negatively associated with central memory T cell, CD4 naive T cell, T helper type 2, T helper type 17 and CD8+ T cell (**Figure 7H**). Higher infiltration of natural regulatory T cells and gamma delta T cells was observed in the SNV gene set than in the WT gene set (**Figure 7I**). In addition, higher infiltration of dendritic cells, natural regulatory T cells, macrophages, monocytes, T helper type 1 cells, and B cells, induced regulatory T cells and the overall infiltration score of 24 immune cells were detected in the CNV gene set than in the WT gene set (**Figure 7J**). Notably, patients with higher risk scores were associated with significantly lower levels of neutrophils, NK cells, T helper type 1, and mast cells (**Figures 7K–N**).

**Figure 8:**
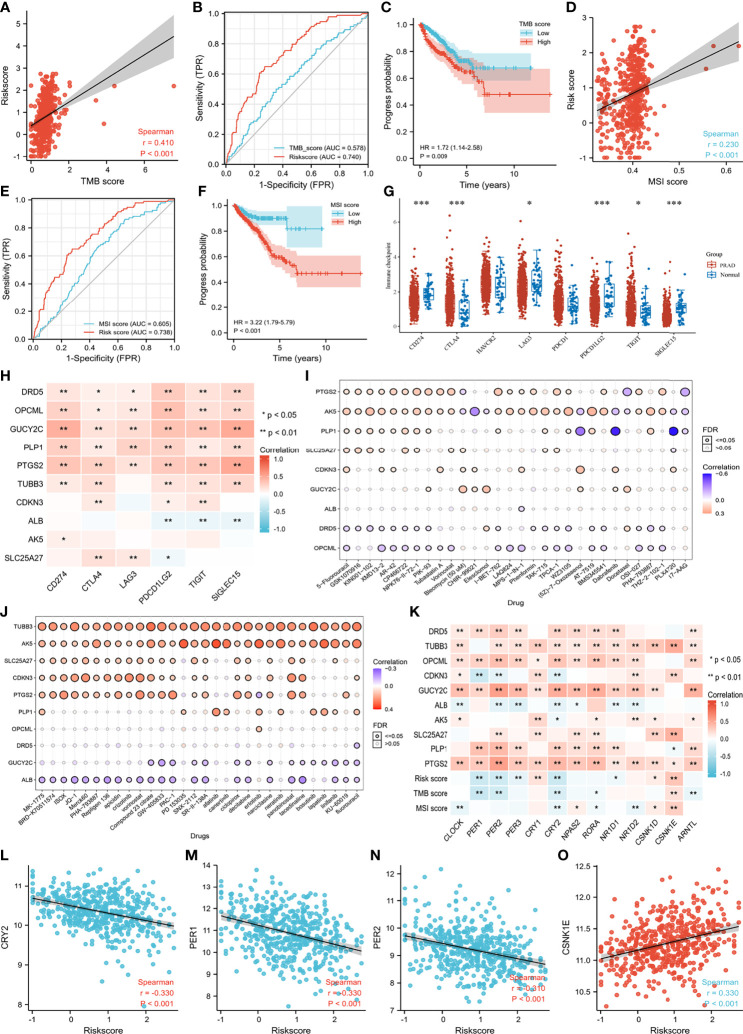
Immune related scores, drug sensitivity and correlation regarding genes of circadian clock pathway. **(A)** the correlation between TMB score and risk score; **(B)** the receiver operating characteristic curves of TMB score and risk score; **(C)** the progress difference of high TMB group and low TMB group; **(D)** the correlation between MSI score and risk score; **(E)** the receiver operating characteristic curves of MSI score and risk score; **(F)** the progress difference of high MSI group and low MSI group; **(G)** the immune checkpoint difference between PCA and normal samples; **(H)** the correlation between enrolled genes and immune checkpoint genes; **(I)** the correlation between gene expression and the sensitivity of GDSC drugs (top 30) in pan-cancer; **(J)** the correlation between gene expression and the sensitivity of CTRP drugs (top 30) in pan-cancer; **(K)** the correlation between genes of circadian clock pathway and enrolled genes and immune scores; **(L)** the correlation between CRY2 and risk score; **(M)** the correlation between PER1 and risk score; **(N)** the correlation between PER2 and risk score; **(O)** the correlation between CSNK1E and risk score. ***means smaller than 0.001.

Of particular concern was the relationship among genes, TMB and MSI. We found that CDKN3 mRNA expression was positively related to TMB score (**Supplementary Figure 3**). Negative correlations were observed between the mRNA expression of GUCY2C and PLP1 and the TMB score (**Supplementary Figure 3**). There was a positive correlation between the risk score and TMB score (**Figure 8A**), Patients and the diagnostic accuracy of risk score was higher than TMB score for PCA progression (**Figure 8B**). with higher TMB scores were more prone to progress than those with lower TMB scores (**Figure 8C**). Likewise, we observed similar results regarding the correlation between the MSI score and risk score and the impact of the MSI score on PFI (**Figures 8D–F**). Furthermore, the expression distribution of immune checkpoint genes in tumor tissues and normal tissues in PCA patients was determined, and we found that CD274 (also named PD-L1), LAG3, PDCD1LG2 (also named PD-L2), TIGIT and SIGLEC15 were significantly downregulated, while CTLA4 was significantly upregulated (**Figure 8G**). We subsequently analyzed the correlations between genes and these immune checkpoint genes. We observed that anti-oncogenes presented a significantly positive correlation with PD-L1, PD-L2, TIGIT and SIGLEC15 (**Figure 8H**). The correlations between gene expression and drug sensitivity in the pancancer analysis of GDSCs and CTRP are shown in **Figure 8I** and **Figure 8J**, respectively. 5-Fluorouracil, GSK1070916, KIN001-102, XMD13-2, MK-1775, BRD-K70511574, ISOX, JQ-1, Merck60, PHA-793887, Repligen 136, apicidin, crizotinib, vorinostat, Compound 23 citrate, GW-405833, and PAC1-1 showed relatively good effects.

CLOCK, PER (1, 2, 3), CRY2, NPAS2, RORA, and ARNTL showed a higher correlation with anti-oncogenes, while CSNK1D and CSNK1E presented a greater relationship with oncogenes (**Figure 8K**). Overall, patients with higher risk scores showed lower mRNA expression of PER1, PER2, and CRY2 and higher expression of CSNK1E (**Figures 8L–O**). There was a positive correlation between MSI score and CSNK1E (**Supplementary Figure 3**). In addition, a negative correlation was observed between TMB score and PER (1,2) and CRY2 (**Supplementary Figure 3**). Specifically, OPCML mRNA expression showed a significantly positive relationship with PER2, CRY2, and RORA. GUCY2C tended to rise with increasing CLOCK, PER2, PER3, CRY2, NPAS2, and RORA (**Supplementary Figure 3**). An obvious positive correlation was observed between PLP1 mRNA expression and PER1 and PER2 (**Supplementary Figure 3**). We detected a significantly positive relationship between CLOCK, PER (1,2,3), CRY2, NPAS2, RORA, ARNTL and PTGS2 mRNA expression (**Supplementary Figure 3**). In contrast, TUBB3 showed an increased tendency with the upregulation of CSNK1D and CSNK1E. SLC25A27 mRNA expression rose with increasing CSNK1E (**Supplementary Figure 3**).

## Discussion

CDH is a pervasive trait of life driven by the molecular CIC system, which constitutes the evolutionary molecular machinery that governs physiological time regulation to preserve homeostasis (13). Prior studies have noted the strong relationship between CIC disruption and cancers, such as breast cancer and colorectal cancer (48, 49). Male androgen secretion is also associated with circadian variations. Significant decreases in total and free testosterone, dehydroepiandrosterone sulfate and mean 24-hour hormonal levels were detected in the elderly population compared to young men (50). Statistically significant CDH was observed in both young and elderly populations for free testosterone levels, while only young men showed a significant CDH for total testosterone and dehydroepiandrosterone sulfate. In addition, the total and free testosterone levels showed the same acrophase at approximately 8 o’clock among young men. However, for the older population, the peak time of free testosterone secretion was at approximately 4 o’clock (50). Thus, CIC desynchronization might be related to the development of PCA. Currently, there is little published information on the role of CIC in PCA. In this study, from the fresh perspective of CIC, we identified ten prognosis-related genes (SLC25A27, ALB, CDKN3, TUBB3, AK5, DRD5, PLP1, PTGS2, OPCML, GUCY2C) that could classify PCA patients undergoing radical prostatectomy in the TCGA database into two groups and first provided a novel nomogram to predict progression for such patients. Among the ten identified genes, ALB (51, 52), CDKN3 (53), TUBB3 (54, 55), DRD5 (56, 57), PTGS2 (58), and OPCML (59, 60) were reported to be associated with the prognosis of PCA, while the potential roles of SLC25A27, AK5, PLP1, and GUCY2C in PCA remained largely unexamined.

Immune cells in the tumor microenvironment play vital roles in tumorigenesis, where antitumor immune cells within the tumor microenvironment tend to kill cancer cells in the early stage of tumorigenesis, but cancer cells appear to eventually evade immune surveillance and even inhibit the cytotoxic effects of antitumor immune cells through a wide spectrum of mechanisms (61). In this study, we observed higher immune scores and higher numbers of macrophages, T cells and myeloid dendritic cells in tumor samples than in normal samples through three algorithms, which indicated the occurrence of immune remodeling during the carcinogenesis of PCA. Neutrophils and NK cells contribute to the killing effects on cancer cells (61). However, both neutrophils and NK cells were negatively associated with the risk score in this study, which promoted progression in high-risk patients. Despite the considerable enthusiasm of immunotherapy for various cancers in the last few years, it seems that combination treatments, including cancer vaccines, checkpoint inhibitors with different immunotherapeutic agents, hormonal therapy (enzalutamide), radiotherapy (radium 223), DNA-damaging agents (olaparib), and chemotherapy (docetaxel), are more promising than immunotherapy alone for PCA outcomes (62). Furthermore, a previous study found that an association between higher TMB and improved survival was observed in patients with bladder cancer receiving immune checkpoint inhibitor treatments (63). Similarly, MSI was shown to be associated with the response to PD-1 treatments in solid tumors, such as metastatic colorectal cancer, and Keytruda was approved to treat MSI-high or mismatch repair-deficient patients with solid tumors (64–67). For PCA patients, a previous study showed that eleven patients with MSI-H/dMMR castration-resistant PCA received anti-PD-1/PD-L1 therapy, among which six patients (54.5%) had a greater than 50% decline in prostate-specific antigen levels, 4 of whom had radiographic responses (68). In this study, we found positive correlations between TMB/MSI and oncogenes and negative correlations between TMB/MSI and anti-oncogenes. Moreover, we observed higher TMB/MIS in high-risk patients, and patients with higher TMB/MSI were more prone to progress than their counterparts. Furthermore, PD-L1, PD-L2, TIGIT and SIGLEC15 might be potential targets, especially PD-L2, due to having the best correlation with anti-oncogenes. Thus, we hypothesized that higher TMB/MSI patients with PD-L2 might have an improved prognosis for PCA patients undergoing radical prostatectomy.

The CIC machinery generates daily CDH, and the circadian oscillator in mammals is based on interlocked transcription-translation feedback loops (13). BMAL1 can form heterodimers with either CLOCK or NPAS2, which acts redundantly but possesses different tissue specificities. The BMAL1:CLOCK and BMAL1:NPAS2 heterodimers activate a set of genes that possess E-box elements (consensus CACGTG) in their promoters. This confers circadian expression on the genes. The PER genes (PER1, PER2, PER3) and CRY genes (CRY1, CRY2) are among those activated by BMAL1:CLOCK and BMAL1:NPAS2. PER and CRY mRNA accumulate in the morning, and the proteins accumulate in the afternoon. PER and CRY proteins form complexes in the cytosol. These complexes can be bound by either CSNK1D or CSNK1E kinases that can phosphorylate PER and CRY. The phosphorylated PER : CRY:kinase complex is translocated into the nucleus due to the nuclear localization signal of PER and CRY. In the nucleus, the PER : CRY complexes bind BMAL1:CLOCK and BMAL1:NPAS2, inhibiting their transactivation and phosphorylation activity, thus reducing the expression of the target genes of BMAL1:CLOCK and BMAL1:NPAS2 in the afternoon and evening (12, 13, 69). PER: CRY complexes also traffic out of the nucleus into the cytosol due to the nuclear export signal of PER. At night, PER : CRY complexes are polyubiquitinated and degraded, allowing the cycle to begin again. Transcription of the BMAL1 gene is controlled by RORA and REV-ERBA, both of which are targets of BMAL1: CLOCK/NPAS2 in mice and compete for the same element (RORE) in the BMAL1 promoter. RORA activates the transcription of BMAL1; REV-ERBA represses the transcription of BMAL1. This mutual control forms a secondary control, thus reinforcing the CIC loop. REV-ERBA shows strong circadian rhythmicity and confers circadian expression on BMAL1 (12, 13, 69).

The CIC is cell-autonomous. Some, but not all, cells of the body exhibit CDH in metabolism, cell division, and gene transcription. The SCN in the hypothalamus is the major clock in the body that receives its major input from light (via retinal neurons) and a minor input from nutrient intake. The SCN and other brain tissues determine waking and feeding cycles, influencing the clocks in other tissues through hormone secretion and nervous stimulation processes. Independent of the SCN, other tissues, such as the liver, receive inputs from nutrients and signals from the brain (12, 13, 69). In this study, we observed that CLOCK, PER (1,2,3), CRY2, RORA, NR1D1 and ARNTL were significantly downregulated, while CSNK1E and CSNK1D were significantly upregulated in PCA patients (**Supplementary Material**). Therefore, CDH disruption might play a significant role in the pathogenesis of PCA. In addition, we observed that oncogenic processes, including the activation of oncogenes and the inhibition of anti-oncogenes, might directly weaken CDH. In high-risk PCA patients, the mRNA expression of PER1, PER2, and CRY2 decreased, while CSNK1E expression increased. This shortened the CIC period, and decreased levels of PER 1 and PER2 promoted androgen excess *via* insulin-like growth factor-binding protein 4 and sex hormone binding globulin in the liver (70). In addition, CRY2 downregulation contributed to the upregulation of PCA (7). Androgens could regulate a number of cellular processes, such as proliferation and signal transduction, mainly through binding to AR (5, 71). The activation of AR signaling dictates the growth of PCA cells and has been thought of as a key factor in PCA tumorigenesis and the progression to androgen-independent PCA (5, 71). Steroid synthesis from the adrenal gland, AR overexpression, and ligand-independent activation of AR by growth factors, cytokines, and steroids other than androgens contributed to the activation of AR despite the lack of androgens (5). Therefore, androgen excess and AR overexpression promoted tumorigenesis and progression in PCA patients with high-risk scores. Similarly, for patients engaged in night-shift work, the synthesis of PER1, PER2 and CRY2 will not decrease as long as retinal neurons receive light signals continuously, and the expression of androgen and AR will not increase thereby. Thus, the PCA risk does not increase significantly, which is consistent with the results of previous epidemiological studies (19, 20). Indeed, the chronic CDH disorder caused by staying up or night-shift work might increase PCA risk through other factors, such as metabolic syndrome and inflammatory bowel disease (72–77).

There can be no denying that gene expression signatures are subject to sampling bias caused by intratumor genetic heterogeneity. In addition, the microenvironment features might be distinct in different tumor regions, such as the tumor core and invasive margin. More importantly, all findings in this study warrant further external validation through large sample research, thereby exploring the potential mechanism of pathogenesis between CIC and PCA.

## Conclusions

We identified ten prognosis-related genes as a promising tool for risk stratification in PCA patients from the fresh perspective of CIC.

## Data Availability Statement

The datasets presented in this study can be found in online repositories. The names of the repository/repositories and accession number(s) can be found in the article/**Supplementary Material**.

## Author Contributions

Conception and design: DF. Administrative support: JA, LY, and WW. Provision of study materials or patients: DF, QX, and FZ. Collection and assembly of data: DF and QX. Data analysis and interpretation: DF and QX. Manuscript writing: All authors. All authors contributed to the article and approved the submitted version.

## Funding

This program was supported by the National Natural Science Foundation of China (Grant Nos. 81974099, 82170785, 81974098, 82170784), programs from Science and Technology Department of Sichuan Province (Grant Nos. 21GJHZ0246), Young Investigator Award of Sichuan University 2017 (Grant No. 2017SCU04A17), Technology Innovation Research and Development Project of Chengdu Science and Technology Bureau (2019-YF05-00296-SN), Sichuan University–Panzhihua science and technology cooperation special fund (2020CDPZH-4). The funders had no role in study design, data collection or analysis, preparation of the manuscript, or the decision to publish.

## Conflict of Interest

The authors declare that the research was conducted in the absence of any commercial or financial relationships that could be construed as a potential conflict of interest.

## Publisher’s Note

All claims expressed in this article are solely those of the authors and do not necessarily represent those of their affiliated organizations, or those of the publisher, the editors and the reviewers. Any product that may be evaluated in this article, or claim that may be made by its manufacturer, is not guaranteed or endorsed by the publisher.
