# Supplementary material for: Identification of a Novel Nomogram to Predict Progression Based on the Circadian Clock and Insights Into the Tumor Immune Microenvironment in Prostate Cancer
Source: Front Immunol. 2022 Jan 27;13:777724. doi: 10.3389/fimmu.2022.777724 (PMC8829569; doi:10.3389/fimmu.2022.777724)

| Characteristics | Total(N) | Univariate analysis | |  | Multivariate analysis | |
| --- | --- | --- | --- | --- | --- | --- |
|  |  | Hazard ratio (95% CI) | P value |  | Hazard ratio (95% CI) | P value |
| T stage | 492 |  |  |  |  |  |
| T2 | 189 | Reference |  |  |  |  |
| T3 | 292 | 3.742 (2.111-6.631) | **<0.001** |  | 1.925 (1.010-3.668) | **0.047** |
| T4 | 11 | 4.879 (1.600-14.883) | **0.005** |  | 2.680 (0.847-8.485) | 0.094 |
| PSA(ng/ml) | 442 |  |  |  |  |  |
| <4 | 415 | Reference |  |  |  |  |
| >=4 | 27 | 4.196 (2.095-8.405) | **<0.001** |  | 2.270 (1.075-4.794) | **0.032** |
| Race | 484 |  |  |  |  |  |
| Asian | 12 | Reference |  |  |  |  |
| Black or African American | 57 | 0.317 (0.097-1.033) | 0.057 |  |  |  |
| White | 415 | 0.535 (0.196-1.465) | 0.224 |  |  |  |
| Gleason score | 499 |  |  |  |  |  |
| 6 | 46 | Reference |  |  |  |  |
| 7 | 247 | 4.397 (0.595-32.504) | 0.147 |  | 3.919 (0.527-29.125) | 0.182 |
| 8 | 64 | 9.866 (1.290-75.435) | **0.027** |  | 6.938 (0.889-54.136) | 0.065 |
| 9&10 | 142 | 22.428 (3.104-162.056) | **0.002** |  | 13.914 (1.875-103.225) | **0.010** |
| Age | 499 |  |  |  |  |  |
| <=60 | 224 | Reference |  |  |  |  |
| >60 | 275 | 1.302 (0.863-1.963) | 0.208 |  |  |  |
| Zone of origin | 275 |  |  |  |  |  |
| Central Zone | 4 | Reference |  |  |  |  |
| Overlapping / Multiple Zones | 126 | 10462999.068 (0.000-Inf) | 0.997 |  |  |  |
| Peripheral Zone | 137 | 8343180.312 (0.000-Inf) | 0.997 |  |  |  |
| Transition Zone | 8 | 5395594.980 (0.000-Inf) | 0.997 |  |  |  |

Table 1. Univariable and multivariable Cox regression analyses

Figure 1. Lasso variable screening, prognosis analysis, time-dependent receiver operating characteristic curves and subgroup Kaplan-Meier curves.


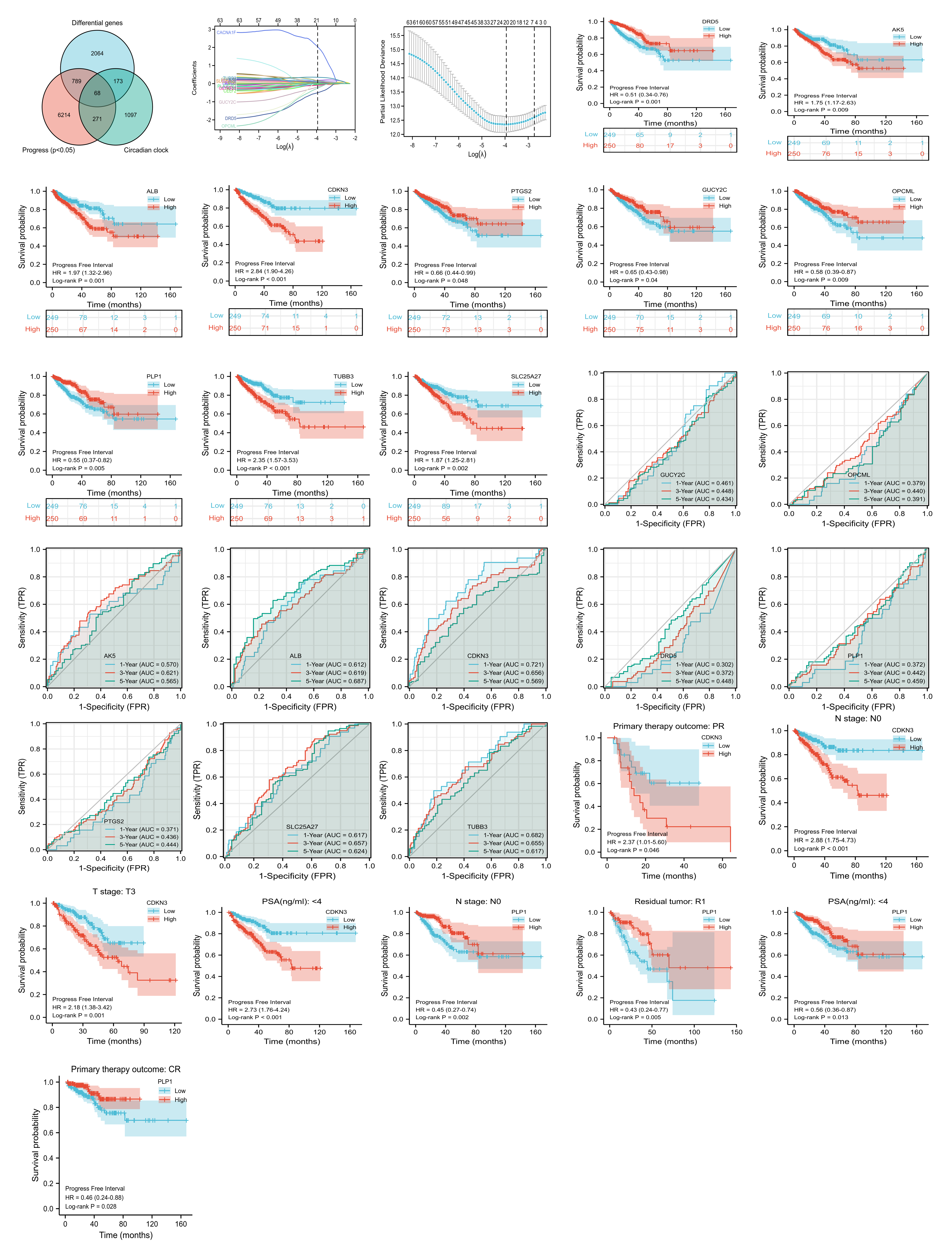


Figure 2 Clinical relevance of enrolled genes in this study.


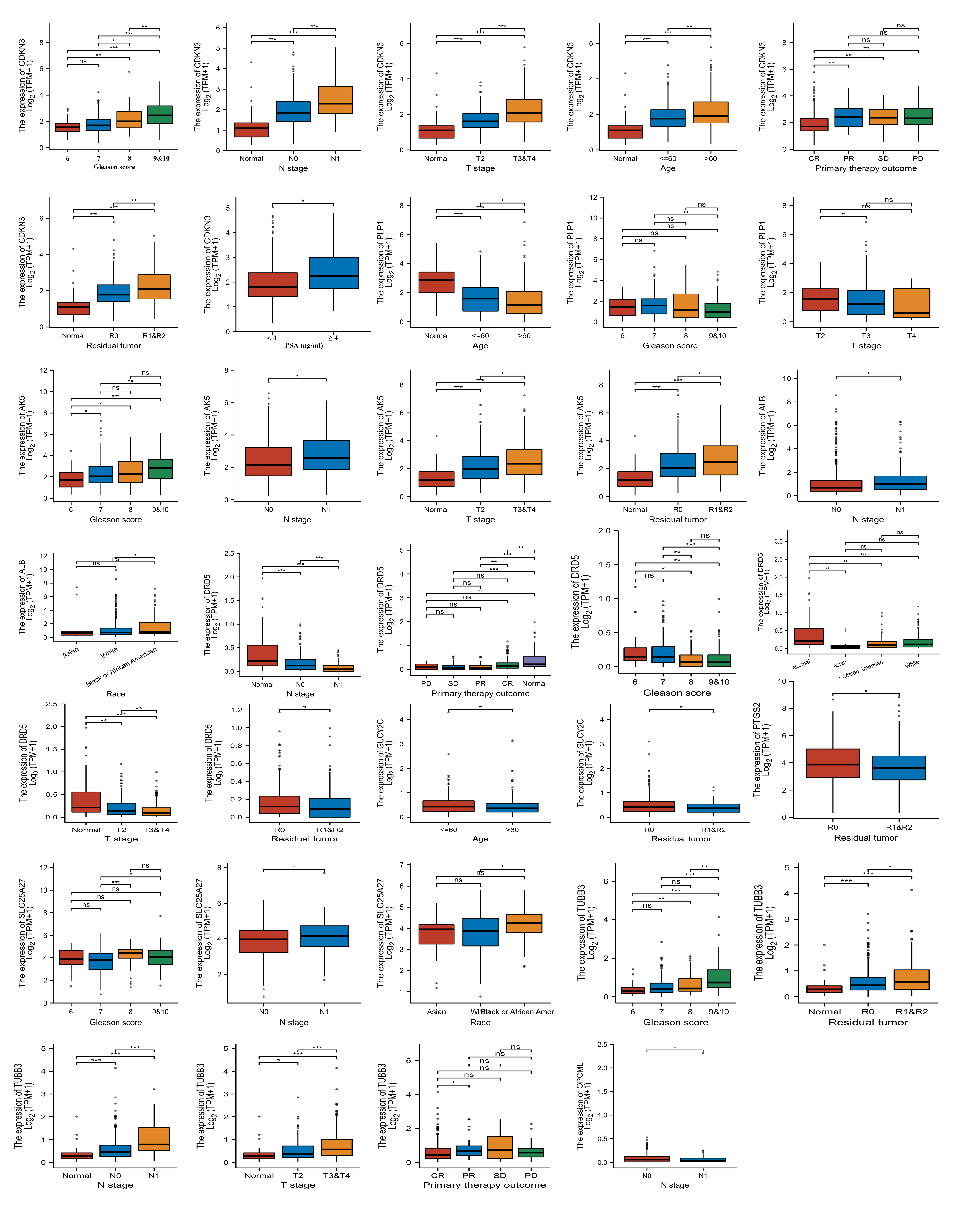


Figure 3. GO, KEGG and GSVA analysis of gene set, relationship between enrolled genes and circadian clock-pathway genes, and mRNA expression of circadian clock-pathway genes in PRAD patients in TCGA database.


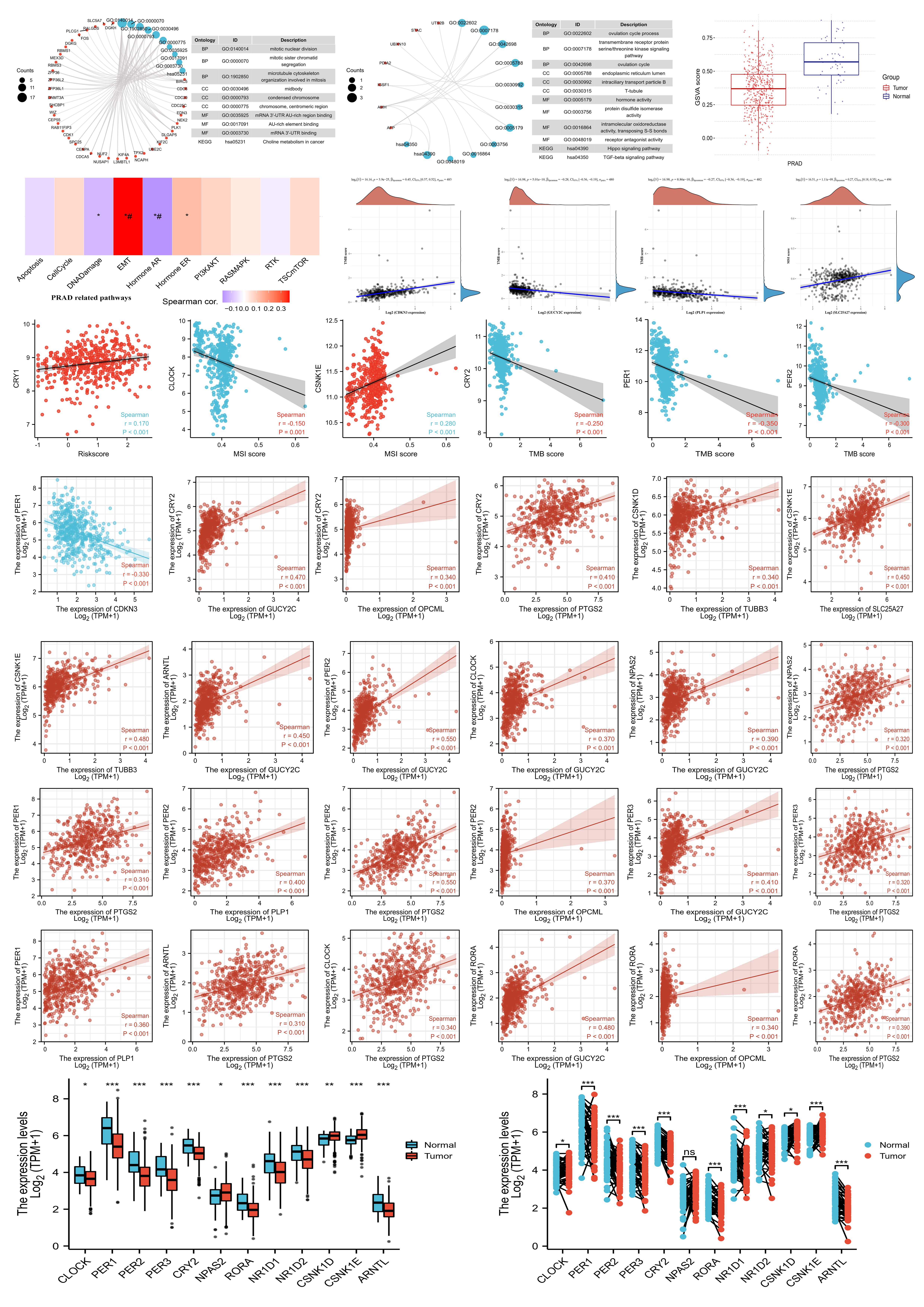

Supplement: Supplementary file 1 [file DataSheet_1.docx]
